# The smell of death: evidence that putrescine elicits threat management mechanisms

**DOI:** 10.3389/fpsyg.2015.01274

**Published:** 2015-08-28

**Authors:** Arnaud Wisman, Ilan Shrira

**Affiliations:** ^1^School of Psychology, University of Kent, Canterbury, UK; ^2^Department of Behavioral Sciences, Arkansas Tech University, Russellville, AR, USA

**Keywords:** olfaction, putrescine, threat, threat management, chemosensory cue

## Abstract

The ability to detect and respond to chemosensory threat cues in the environment plays a vital role in survival across species. However, little is known about which chemical compounds can act as olfactory threat signals in humans. We hypothesized that brief exposure to putrescine, a chemical compound produced by the breakdown of fatty acids in the decaying tissue of dead bodies, can function as a chemosensory warning signal, activating threat management responses (e.g., heightened alertness, fight-or-flight responses). This hypothesis was tested by gaging people’s responses to conscious and non-conscious exposure to putrescine. In Experiment 1, putrescine increased vigilance, as measured by a reaction time task. In Experiments 2 and 3, brief exposure to putrescine (vs. ammonia and a scentless control condition) prompted participants to walk away faster from the exposure site. Experiment 3 also showed that putrescine elicited implicit cognitions related to escape and threat. Experiment 4 found that exposure to putrescine, presented here below the threshold of conscious awareness, increased hostility toward an out-group member. Together, the results are the first to indicate that humans can process putrescine as a warning signal that mobilizes protective responses to deal with relevant threats. The implications of these results are briefly discussed.

## Introduction

When animals die they release an unpleasant smell. A pungent component of this scent is emitted by putrescine, a volatile diamine that results from the breakdown of fatty acids in the putrefying tissue of dead bodies (Hussain et al., 2013). Interestingly, animal research shows that putrescine can function as a powerful chemosensory signal that prompts the perceiver to leave or avoid the area (Yao et al., 2009; Prounis and Shields, 2013). The aim of the present research is to show that humans respond in a similar way to putrescine, and more generally, that exposure to putrescine triggers threat management behaviors (Blanchard et al., 2001; Neuberg et al., 2011).

A growing body of research suggests that humans can identify threats via chemosignals (Chen and Haviland-Jones, 2000; Ackerl et al., 2002; Prehn et al., 2006; Mujica-Parodi et al., 2009; Zhou and Chen, 2009; de Groot et al., 2012). For instance, when people are exposed to sweat taken from donors during a fearful experience, perceivers show a heightened startle reflex (Prehn et al., 2006; Pause et al., 2009) and interpret ambiguous facial expressions as fearful (Zhou and Chen, 2009). This transmission of threat-arousing chemosignals is assumed to serve an adaptive function by orienting us to impending dangers. Indeed, the ability to detect and process chemosensory threat cues is vital for the survival of a wide range of species (Stevenson, 2010). However, thus far there is little evidence that humans can, like other organisms, detect olfactory threat cues in the environment through means other than the chemosignals (e.g., body sweat) of conspecifics.

The decay of tissue and its resulting scent can function as a “necromone” cue that signals an animal’s death to conspecifics. Alarm and avoidance behaviors (necrophobic behaviors) in response to these scents are widespread in the animal kingdom and thought to have evolved at least 420 million years ago (Yao et al., 2009). In fact, recent research shows that necrophobic behavior may have innate underpinnings through the activation of trace amine-associated receptors (TAARs), a group of specialized scent receptors in the olfactory epithelium (Hussain et al., 2013; Horowitz et al., 2014; Li and Liberles, 2015). TAARs are known to detect specific chemicals that evoke behavioral responses, without the need for prior exposure to the scents. For example, in model vertebrates, certain TAARs respond to diamines (e.g., putrescine) by producing avoidant behaviors that likely serve to defend against immediate dangers (Yoon et al., 2015). Thus, it is feasible that we have a chemosensory sensitivity to diamines like putrescine (Li and Liberles, 2015), given that their detection can aid survival (Stevenson, 2010).

A further advantage of examining putrescine as a threat stimulus is that we know what it is. Despite the impressive amount of indirect support for human chemosignals amassed in recent years, their chemical properties have yet to be identified (Wyatt, 2009). Focusing on a known compound, putrescine, enables us to directly test whether it plays a causal role in human threat responses. In a similar vein, although several studies have shown that chemosensory cues can elicit greater readiness for behavior (Bradley et al., 2001; Prehn et al., 2006), thus far there is little direct evidence that a specific chemical substance can cause overt behavioral changes in humans (Wysocki and Preti, 2004). Since exposure to putrescine elicits specific behaviors in animals (e.g., escape, avoidance), we can examine whether putrescine produces similar behaviors in humans. In sum, putrescine appears to be well-suited to test as a specific chemical compound that can act as a threat signal in humans.

Chemosensory cues can convey danger in at least two fitness-relevant domains: microbial and predator threats (Stevenson, 2010). First, olfactory information is often central to identifying the presence of pathogens. For example, pathogens can alter the scent of those who become infected, which can be detected by conspecifics (Arakawa et al., 2010; Tybur et al., 2011; Olsson et al., 2014). Similarly, the release of putrescine in decaying tissue co-occurs with the arrival of bacteria, a motivation for others to eschew physical contact with the dead body. A number of species exhibit necrophobic behaviors, and after detecting the scent emanating from dead bodies, usually respond by leaving or avoiding the area (Prounis and Shields, 2013). Second, putrescine released by decaying bodies can signal the risk of predation (Boissy et al., 1998). Since a large proportion of deaths in the wild are the result of predator attacks, putrescine would be a useful alarm cue to stay away (Misslin, 2003).

In humans, responses to specific scents can develop through learned associations between odors and personal experiences (Stevenson et al., 1998; Degel et al., 2001). For example, based on the cultural expression that when “something smells fishy” it is viewed suspiciously, exposure to fish-like odors arouses suspicion toward others and reduces cooperation, an orientation that is assumed to result from conditioned reactions to this scent (Lee and Schwarz, 2012). Likewise people may learn to associate the smell of putrescine with threats, and it is plausible that occasional exposure to putrescine, whenever it occurs, could lead to conditioned threat responses (Stevenson, 2010). However, we render it unlikely that modern humans have strong conscious associations with the scent of putrescine. Moreover, conscious scent evaluations are often inaccurate, context dependent, and colored by other sensory modalities (Sela and Sobel, 2010). In view of this, it is important to note that responses to aversive chemosensory cues do not require prior learning or conscious evaluation (Dielenberg et al., 2001; Miller and Maner, 2010; Li et al., 2007). Indeed, scents can alter our perception, cognition, behavior, and physiology (e.g., heart rate, skin conductance) even when there is no conscious scent detection (Li et al., 2007; Pause et al., 2009; Sela and Sobel, 2010; Krusemark and Li, 2012), and even after olfactory adaptation has set in (de Groot et al., 2012; Smeets and Dijksterhuis, 2014). Thus, neither prior associations with olfactory signals, nor conscious processing, are necessary conditions for people to process them as threatening (Köster et al., 2002; Williams et al., 2006; Sela and Sobel, 2010; Pause, 2012; Smeets and Dijksterhuis, 2014).

At the most basic level, threat detection increases vigilance and sharpens our reactions to events in the environment (Williams et al., 2006). For instance, detection of a predator’s scent will interrupt foraging and increase behaviors (e.g., scanning the environment) that facilitate predator detection (Woody and Szechtman, 2011). Once the threat management system is engaged, it produces readiness for fight-or-flight behaviors (Cannon, 1927; Blanchard et al., 1986; Gray and McNaughton, 2003; Mobbs et al., 2009). Flight responses seek to escape the situation, whereas fight responses—whether physical or verbal aggression—are typically only used when escape is not possible. In contrast to popular belief that the dominant response to threats is to fight, flight is actually far more common (Misslin, 2003), presumably because nature selects more strongly for strategies that minimize risk. In one study, for example, when people were confronted by a threatening out-group member, they responded with aggressive readiness (fight), but only when there was little possibility of escaping; when given the option, though, participants chose to distance themselves (flight) from the other person (Cesario et al., 2010).

## Overview and Hypotheses

Coming full circle, we propose that putrescine can serve as a (non-conscious) signal that initiates threat management responses. Specifically, we hypothesize that brief exposure to putrescine increases vigilance, followed by the readiness to either escape (flight), or engage in aggressive readiness (fight) when escape is not possible. Experiment 1 assessed whether putrescine (vs. ammonia and a neutral scent) increased vigilance as measured by faster responses in a simple reaction time task. Experiments 2 and 3 assessed whether brief exposure to putrescine (vs. ammonia and neutral scent) caused participants to walk away faster from the exposure site after completing the experiment (outdoors). Experiment 3 also tested whether putrescine evoked cognitions related to escape and threat. Finally, Experiment 4 examined whether non-conscious exposure to putrescine increased aggressive readiness (e.g., defensiveness toward an out-group member). All four experiments adhered to the Declaration of Helsinki guidelines, and gained the prior approval by the University Research Ethics Committee. Written consent was obtained from all participants involved in these experiments, and all were fully debriefed.

## Experiment 1: The Effect of Putrescine on Vigilance

In Experiment 1, we tested whether brief exposure to putrescine increased vigilance. To measure vigilance, we employed a task closely modeled after the shortened version of the psychomotor vigilance task (PVT; Dinges and Powell, 1985) that assessed participants’ reaction times to a red dot that was presented at random intervals on a computer screen.

In addition, Experiment 1 was designed to determine whether ammonia served as an appropriate aversive control condition. Our pilot testing revealed that ammonia, unlike other aversive scents we had examined (i.e., skatole^1^ and indole), was rated similarly to putrescine on repugnance, familiarity, and intensity. Moreover, previous research has used ammonia (NH_3_; ammonium hydroxide) as an aversive scent prime (Rieser et al., 1976; Wise et al., 2005) and ammonia can increase trigeminal nerve activation associated with vigilance and sensory rejection, via activation of the sympathetic nervous system (Hummel and Kobal, 1992; Sekizawa and Tsubone, 1994). However, some research suggests that unpleasant ambient odors can also decrease reaction times on simple tasks like the PVT (Millot et al., 2002). In view of this, we made no specific prediction about whether ammonia, like putrescine, would enhance vigilance relative to our scentless control condition.

### Method

#### Participants and Procedure

A sample of 60 participants (43 females; *M*_*age*_ = 21.20, SD = 3.20) completed the study in return for a financial incentive of 3£ (approximately $5).

Participants were randomly assigned to one of three conditions: putrescine (C_4_H_12_N_2_; Sigma-Aldrich), ammonia (5%; NH_3_; Sigma-Aldrich), or water. One hour before the start of the experiment, cotton wool pads were blotted with 2 ml of one of the three compounds, and stored separately in small (100 ml) sealable amber jars. Participants were run in our lab individually, and seated in different cubicles to avoid carryover effects of scents. The refreshment rate in each cubicle was 4–5 air changes (cycles) per hour. Furthermore, participants were booked at least 30 min apart in order to ventilate the rooms—by opening the lab room’s window—between sessions. When preparing materials for the experiment, one of the researchers marked the bottom of each jar with a number code, so that the experimenters were unaware of the meaning of these codes. This basic procedure was repeated in our subsequent experiments to keep the experimenters blind to the conditions.

Participants were seated in front of a standard PC (equipped with Authorware 7.1 software) with a 17-inch screen. They were given instructions (on-screen) to open the jar, sniff the scent inside for 10 s, and close the jar. After that, they rated the scent on its intensity (“This scent is intense”; 1 = *strongly disagree* and 9 = *strongly agree*), repugnance (“This scent is repugnant”; 1 = *strongly disagree* and 9 = *strongly agree*), and familiarity (“This scent is familiar”; 1 = *strongly disagree* and 9 = *strongly agree*). Repugnance was included as evaluative rating (alongside the standard measures of intensity and familiarity) because repugnance (or disgust) is often a central component of aversive scents. Participants were then introduced to the adapted PVT, which lasted about 5 min (see Loh et al., 2004). The task instructed them to click on a red dot as quickly as possible whenever they saw the dot on the screen. Ten dots (each measuring 1 cm) were shown at different locations on the screen, and the time between appearances was randomized at variable intervals (2–45 s). As soon as participants clicked on the red dot with the mouse, a screen appeared for 5 s with the message: “prepare for next trial.” Participants received two practice trials first, to get them familiar with the main task of 10 trials. Finally, after completing the PVT and filling out a standard demographic questionnaire, they were fully debriefed and thanked for their participation.

### Results and Discussion

#### Hedonic Evaluations

We began by testing our prediction, based on our pilot testing, that putrescine and ammonia would not differ from each other on repugnance, familiarity and intensity. As predicted, separate one-way between-subjects ANOVAs revealed that there was no significant difference between ammonia and putrescine on repugnance, *F*(1,38) = 0.38, *p* = 0.54, η^2^ = 0.01, familiarity, *F*(1,38) = 0.26, *p* = 0.26, η^2^ = 0.03, or intensity, *F*(1,38) = 0.14, *p* = 0.71, η^2^ = 0.004 (see Table 2, for descriptive statistics). Moreover, the analyses reported below were not altered when entering all hedonic evaluations as covariates.

**TABLE 2:** **Scent ratings for the chemosensory primes (Experiment 1)**.

**Chemosensory primes**	**Neutral**	**Ammonia**	**Putrescine**
Intensity			
*M*	3.30	4.73	4.27
SD	1.81	1.45	1.92
Familiarity			
*M*	6.00	5.10	4.40
SD	0.86	2.25	1.60
Repugnance			
*M*	2.35	5.90	5.65
SD	1.46	1.34	1.23
*N*	20	20	20

#### Reaction Times

We examined our main prediction that putrescine, relative to the neutral control condition (water), would elicit faster reaction times. In line with previous PVT research, we applied reciprocal transformation to the raw data (i.e., 1/RT). This type of transformation is standard within the PVT paradigm, as it reduces the impact of extreme scores and brings them into an acceptable range (Dinges et al., 1987; Dorrian et al., 2004). A one-way between-subjects ANOVA revealed a difference between the scent conditions, *F*(2,57) = 4.32, *p* = 0.018, η^2^ = 0.13. *Post hoc* comparisons, with the raw means reported here, showed that putrescine produced faster reaction times (*M* = 1.04, SD = 0.10) than the neutral scent (*M* = 1.24, SD = 0.35; *p* = 0.013), but not compared to ammonia (*M* = 1.12, SD = 0.20; *p* = 0.28). No difference was found between the neutral and ammonia conditions (*p* = 0.14).

In sum, only putrescine caused participants to react more quickly compared to the neutral condition, supporting our hypothesis that putrescine increases vigilance. At the same time, ammonia did not increase vigilance relative to the scentless control condition. Importantly, the findings show that, consistent with our pilot study, ammonia and putrescine were evaluated similarly on repugnance, familiarity, and intensity, and were similar in the degree of vigilance they elicited. Consequently, together with previous research (Rieser et al., 1976; Wise et al., 2005), Experiment 1 indicated that ammonia would serve as an appropriate aversive control condition. Experiments 2 and 3 investigated our hypothesis that putrescine activates the motivation to escape the situation (flight).

## Experiment 2: The Effect of Putrescine on Escape Behavior

Similar to Experiment 1, Experiment 2 first asked participants to rate a scent prime (putrescine vs. ammonia vs. neutral) on three dimensions: intensity, familiarity, and repugnance, then we observed whether it influenced the tendency to escape the situation. To avoid the biases associated with some operationalizations of flight in prior research (e.g., self-reported intentions, Gilbert and Gilbert, 2003), we employed an overt behavioral measure of escape (e.g., Ellsworth et al., 1972; Wisman and Koole, 2003). Specifically, we assessed whether putrescine would cause participants (who were under the impression the study was finished) to walk away more quickly over a predetermined distance of 80 m.

### Method

#### Participants and Procedure

Forty-five participants (21 females and 24 males; *M*_*age*_ = 27.51, SD = 9.72) completed the study on campus. We filled three empty felt-tip pens, each with one of the three compounds (putrescine, ammonia, or water). To fill each pen, 10 ml of liquid odor was injected onto the pen’s fiber rod inside the pen. The pens were then re-assembled and left to stand upside down for 24 h in order to allow the liquid to soak into the fiber rod. Just before the start of the experiment, scent blotters were marked with the scent marker pens and stored in separate sealable containers.

Participants were approached on a fixed spot on the campus and asked if they had time to participate in a brief scent test of approximately 10 min. Participants were tested individually and randomly assigned to one of three conditions (putrescine, ammonia, or water). The experimenter, blind to the conditions, presented one of the three containers to the participant, who rated the scent on intensity (“This scent is strong”; 1 = *strongly disagree* and 5 = *strongly agree*), repugnance (“This scent is repugnant”; 1 = *strongly disagree* and 5 = *strongly agree*), and familiarity (“This scent is familiar”; 1 = *strongly disagree* and 5 = *strongly agree*). After finishing and being thanked for their participation, a second experimenter—blind to the condition and hypotheses of the experiment, and out of sight of the participants—used a standard stopwatch to time how many seconds it took participants to walk away over a distance of 80 m (pre-measured before the experiment began). The recorded time constituted our dependent variable. After they reached this distance, participants were re-approached, fully debriefed and thanked again.

### Results and Discussion

#### Hedonic Evaluations

Consistent with Experiment 1, separate one-way between-subjects ANOVAs revealed that there was no significant difference between ammonia and putrescine on repugnance, *F*(1,28) = 2.30, *p* = 0.14, η^2^ = 0.07, and familiarity, *F*(1,28) = 0.04, *p* = 0.75, η^2^ = 0.01. However, ammonia was rated as relatively more intense (*M* = 4.73; SD = 0.46) compared to putrescine (*M* = 4.27; SD = 0.70; *p* = 0.04; see Table 3). Once again, the results reported below were not altered when we entered the intensity (nor the other hedonic) ratings into the analyses as covariates. We also note that the results were similar whether participants rated how “intense” or “strong” the scent smelled (see Experiment 3 below).

**TABLE 3:** **Scent ratings for the chemosensory primes (Experiment 2)**.

**Chemosensory primes**	**Neutral**	**Ammonia**	**Putrescine**
Intensity			
*M*	1.53	4.73	4.27
SD	0.64	0.46	0.70
Familiarity			
*M*	4.75	1.60	1.67
SD	0.46	0.51	0.62
Repugnance			
*M*	1.73	4.47	4.80
SD	0.70	0.74	0.41
*N*	15	15	15

#### Escape Behavior

**FIGURE 1:**
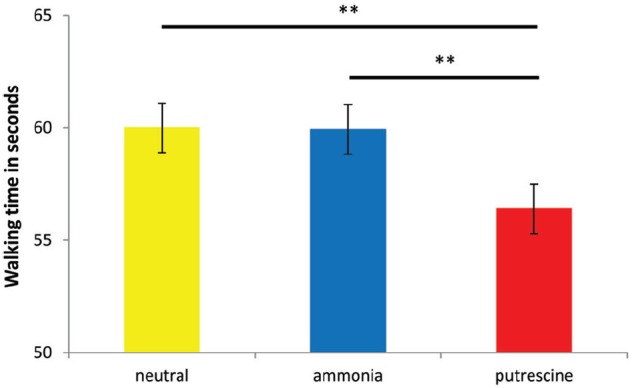
**The number of seconds it took participants to walk 80 m after exposure to the scent prime (Experiment 2).** Asterisks denote that two groups differ at **p < 0.005.

To test our hypothesis that putrescine elicited an escape motivation, we compared our scent conditions in a one-way ANOVA, using gender as a covariate^2^. The results yielded a significant effect of the scent prime on the time it took to walk 80 m, *F*(2,41) = 19.03, *p* < 0.001, η^2^ = 0.48. The only significant differences occurred between putrescine (*M* = 56.40 s; SD = 4.19) and ammonia (*M* = 59.93, SD = 5.04), and between putrescine and the neutral scent prime (*M* = 60.00, SD = 4.42; both *p*s < 0.005; see Figure 1). Thus, putrescine caused participants to walk away more quickly, supporting our assumption that putrescine evoked a stronger motivation to escape. Experiment 3 was conducted to replicate this finding, and furthermore to test whether putrescine elicited implicit cognitions related to escape and threat.

## Experiment 3: The Effect of Putrescine on Escape Behavior and Thoughts

The procedure for Experiment 3 was similar to Experiment 2’s. First, we asked participants to evaluate the scents on the different dimensions (repugnance, familiarity, intensity). In addition, we gaged participants’ implicit threat-related associations using a word stem-completion task. Specifically, this task measured the implicit accessibility of thoughts related to “escape” and “threat.” We predicted that only putrescine would increase the accessibility of these cognitions. Finally, we assessed whether putrescine would cause participants to walk away more quickly over a predetermined distance of 60 m.

### Method

#### Participants and Procedure

Sixty participants (32 females and 28 males, *M*_*age*_ = 21.57, SD = 1.12) completed the study on campus. Individuals were approached just outside campus on a path sloping downhill and asked if they had time to participate in a brief scent test for about 15 min.

Participants were randomly assigned to one of the three scent conditions, then they rated the scent on intensity, repugnance, and familiarity (“This scent is intense”; 1 = *strongly disagree* and 9 = *strongly agree*), repugnance (“This scent is repugnant”; 1 = *strongly disagree* and 9 = *strongly agree*), and familiarity (“This scent is familiar”; 1 = *strongly disagree* and 9 = *strongly agree*). Then, to assess cognitions relevant to the concepts of “escape” and “threat,” participants completed the word-stem completion task, a widely used and well-established measurement that gaged the thought accessibility of these two concepts (Greenberg et al., 1994; Arndt et al., 1997; Lozito and Mulligan, 2010; Migo et al., 2010). Participants were asked to complete 30 word fragments, 20 of which were neutral (e.g., B_ NK could be BANK or BUNK) in terms of any particular theme, five of which could be words related to “escape” (e.g., the fragment RU_ could be completed as RUN or RUB, the latter a neutral word), and another five could be completed with a word related to “threat” (e.g., _ _ RROR could be TERROR or MIRROR). We summed the number of escape- (*M* = 2.73, SD = 1.07) and threat-related words (*M* = 1.90, SD = 0.66) that participants completed to assess the thought accessibility of these concepts. Finally, participants were again timed by a second experimenter, who was blind to the conditions and the hypotheses, for how long it took them to walk away over a distance of 60 m (due to natural constraints a slightly shorter distance was used than in Experiment 2).

### Results and Discussion

#### Hedonic Evaluations

Separate one-way between-subjects ANOVAs revealed no difference between the chemosensory primes on repugnance, *F*(1,38) = 0.35, *p* = 0.56, η^2^ = 0.01, familiarity, *F*(1,38) = 0.04, *p* = 0.85, η^2^ = 0.001, and intensity, *F*(1,38) = 0.29, *p* = 0.59, η^2^ = 0.008 (see Table 4). Thus, participants rated ammonia and putrescine similarly to one another on each dimension. Again, the results reported below were did not differ when we entered the hedonic evaluations into the analyses as covariates.

**TABLE 4:** **Scent ratings for the chemosensory primes (Experiment 3)**.

**Chemosensory primes**	**Neutral**	**Ammonia**	**Putrescine**
Intensity			
*M*	1.85	3.20	3.40
SD	0.99	1.32	0.99
Familiarity			
*M*	2.95	2.20	2.15
SD	0.83	0.89	0.75
Repugnance			
*M*	2.60	3.70	3.50
SD	0.60	0.98	1.15
*N*	20	20	20

#### Escape- and Threat-Related Cognitions

To test our hypothesis that putrescine elicited implicit cognitions related to escape and threat, we analyzed the escape and threat word-completion results separately. The results revealed a significant effect of scent prime on escape thought accessibility, *F*(2,57) = 10.90, *p* < 0.001, η^2^ = 0.28 (see Table 5). Putrescine caused participants to complete word stems more frequently with escape related words (*M* = 3.45, SD = 0.69) than both the ammonia (*M* = 2.45, SD = 1.05) and the neutral scent (*M* = 2.15, SD = 0.99) primes (both *p*s < 0.005). Similarly, the scent primes affected the accessibility of threat-related thoughts, *F*(2,57) = 8.39, *p* < 0.001, η^2^ = 0.23. Putrescine led to more threat word-stem completions (*M* = 2.55, SD = 0.94) than ammonia (*M* = 1.73, SD = 0.64) and the neutral scent (*M* = 1.68, SD = 0.65; both *p*s < 0.005).

**TABLE 5:** **The ratings of escape-related and threat-related cognitions for the chemosensory primes (Experiment 3)**.

**Chemosensory primes**	**Neutral**	**Ammonia**	**Putrescine**
Escape cognitions			
*M*	2.15	2.45	3.45
SD	0.99	1.05	0.69
Threat cognitions			
*M*	1.68	1.73	2.55
SD	0.65	0.64	0.94
*N*	20	20	20

#### Escape Behavior

**FIGURE 2:**
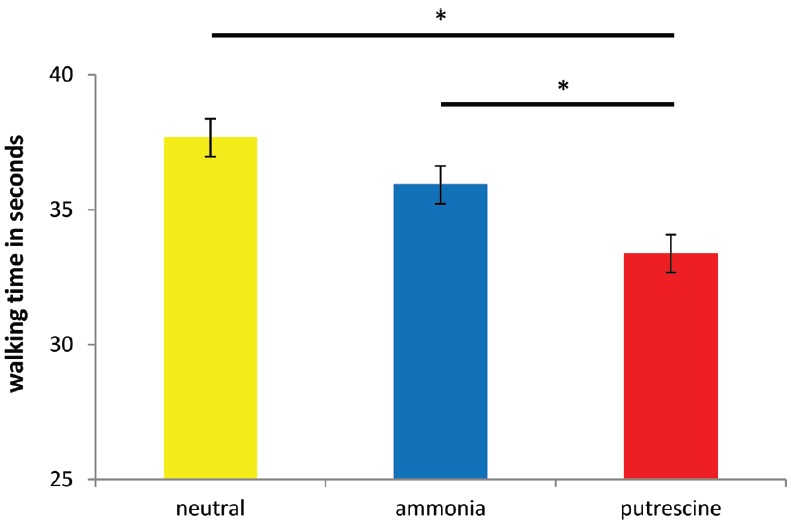
**The number of seconds it took participants to walk 60 m after exposure to the scent prime (Experiment 3).** Asterisks denote that two groups differ at *p < 0.05.

Like Experiment 2, the analyses showed a significant effect of chemosensory primes on walking speed, *F*(2,56) = 9.11, *p* < 0.001, η^2^ = 0.24 (see Figure 2). The pattern of results again showed that putrescine (*M* = 33.38, SD = 2.99) caused people to walk more quickly than ammonia (*M* = 35.92, SD = 3.38) and the neutral scent prime (*M* = 37.67, SD = 3.13; *p* < 0.05). Again, no difference was found between the ammonia and the neutral scent condition (*p* = 0.87).

Experiment 3 revealed that putrescine elicited implicit cognitions of escape and threat. In addition, Experiment 3 replicated the finding that putrescine increased walking speed. Thus, taken together, the results of Experiments 2 and 3 indicated that putrescine motivated (automatic) escape behavior. An important feature of the settings in Experiments 2 and 3 was that participants were outdoors and in a context that facilitated the possibility that they could distance themselves from the scent.

## Experiment 4: The Effects of Putrescine on Defensive Responses Toward An Out-Group

Experiment 4 sought to extend our understanding of the effects of putrescine in two important respects. First, we tested the hypothesis that *non-conscious* (unobtrusive) exposure to putrescine could elicit threat management responses. As we highlighted in the Introduction, this possibility is consistent with evidence that scent primes, even when presented at sub-threshold levels, can influence brain activation (Sobel et al., 1999), learning (Köster et al., 2002), and physiological state (Stern and McClintock, 1998). This applies similarly to aversive scent primes, which for example, have the ability to alter skin conductance (Jacquot et al., 2004), social preferences (Li et al., 2007), and cognitive performance (Epple and Herz, 1999) in ways that correspond to supraliminal exposure to aversive stimuli (Sela and Sobel, 2010). Thus, we predicted that subliminal presentation of putrescine would be capable of activating threat responses.

Second, Experiment 4 focused on the fight rather than the flight component of alarm responses. Consistent with previous research showing that implicit threat cues increase intolerance toward out-group members (Holbrook et al., 2011) and defensive responses (Blanchard et al., 2001; Wheatley and Haidt, 2005), we hypothesized that putrescine would increase defensiveness toward an out-group member, in a situation where there was no immediate opportunity to escape (Cesario et al., 2010). Like Experiment 1, we conducted this experiment in a laboratory setting. After priming the participants with one of the scents, they filled out a standard Positive And Negative Affect Scale (PANAS) that gaged their mood. Although our pilot study (see Table 1) and some research (e.g., Knasko, 1993) revealed that aversive scent primes do not alter mood on a conscious level, we intended to rule out the possibility that the subliminal primes influenced participants’ feelings at a conscious level. After that, they read about an out-group member—a foreign student who criticized the participants’ value system—and were asked to evaluate the target. This evaluation was designed to assess how much hostility participants felt toward the target.

**TABLE 1:** **Hedonic evaluations of putrescine, ammonia, indole, “fart spray,” and skatole^1^ (Pilot study)**.

**Scent primes**	**Putrescine**	**Ammonia**	**Indole**	**Skatole**	**Fart spray**
Intensity^2^					
*M*	5.98_*b*_	6.60_*b*_	5.25_*a*_	7.23_*c*_	5.52_*b*_
SD	2.50	2.46	2.15	2.08	2.07
Familiarity					
*M*	4.98_*a*_	5.10_*a*_	6.88_*b*_	5.21_*a*_	4.90_*a*_
SD	2.71	2.95	2.46	2.56	2.69
Repugnance					
*M*	5.94_*b*_	5.94_*b*_	3.65_*a*_	6.54_*b*_	5.31_*b*_
SD	2.65	2.55	1.78	2.94	2.63
Positivity					
*M*	2.63_*b*_	2.69_*b*_	3.81_*a*_	2.50_*b*_	2.67_*b*_
SD	1.55	1.78	2.05	1.87	1.77
*N*	48	48	48	48	48

*^1^ “How intense is this scent?”, 1 Not at all and 10 Very much; “How familiar is this scent?”, 1 Not at all and 10 Very much; “How repugnant is this scent?”, 1 Not at all and 10 Very much; “How positive does this scent make you feel?”, 1 Not at all and 10 Very much*.

*^2^ Different subscripts on a hedonic dimension (within a row) indicate a significant difference of p < 0.05*.

### Method

#### Participants and Procedure

Sixty-nine participants (39 females and 30 males, *M*_*age*_ = 24.00, SD = 8.38) were run in our lab individually, in different cubicles (randomized) to avoid carryover effects of scents. Furthermore, participants were booked at least 30 min apart in order to ventilate the rooms between sessions. Upon arrival, participants were given the first of two questionnaire packets to complete. This first questionnaire consisted of demographic questions and a number of filler items. We then randomly assigned participants to their condition by marking one of the three liquid scents (putrescine, ammonia, water) to the top of each page (0.5 ml) of the second questionnaire participants were given. In the putrescine and ammonia conditions, this amounted to a very subtle scent prime that was not meant to be detected. At the conclusion of the experiment, we funnel debriefed participants to determine whether they noticed or smelled anything unusual during the study. None of them reported being aware of the scents.

The second questionnaire assessed participants’ mood, and our dependent variables. First, to rule out the possibility that our results could be explained by generalized affect, participants began the second part of the questionnaire by completing the 20-item PANAS (Tellegen et al., 1988). This scale measured the extent to which each of 10 positive affect descriptors (*α* = 0.86) and 10 negative affect descriptors (*α* = 0.85) reflected how they felt at that moment (1 = *very slightly or not at all*, 5 = *extremely*). We computed the average positive affect (*M* = 3.31, SD = 0.68) and negative affect (*M* = 1.61, SD = 0.59) scores for everybody.

This was followed by the description and evaluation of the out-group member (Greenberg et al., 2001; Navarrete et al., 2004; Norenzayan et al., 2007). Specifically, participants read an essay supposedly written by a college student from the Middle East who was visiting the United Kingdom to study English. In this essay, the student went on to criticize Western values, predicting their eventual decline (see Norenzayan et al., 2007). Participants were then asked to evaluate the author and his message by responding to four questions on a 9-point Likert scale [“To what extent do you like the author”; “To what extent would you like to be friends with the author”; “How much would you oppose the author teaching your (future) children”; and “How much do you want the ideas of the author to be publicized”; 1 = *very much*, 9 = *not at all*]. We derived an overall out-group hostility index (*M* = 5.82, SD = 1.63) by averaging all items together (*α* = 0.77), such that larger values indicated greater hostility. Finally, we measured motivation to escape the situation by timing how long it took participants to complete the second (scented) questionnaire followed by a standard demographic questionnaire (91% of the participants were native to England, 3% Greece, 4% Ireland, and 1% to the United States).

### Results and Discussion

#### Ancillary Analyses

One-way ANOVAs tested whether the chemosensory primes elicited different levels of self-reported affect across the three conditions. However, the primes had no impact on positive affect *F*(2,66) = 1.87, *p* > 0.16, nor negative affect, *F*(2,66) = 0.36, *p* > 0.70. Moreover, the analyses below were no different when we used these affect measures as covariates, showing that any effect of our primes on out-group defense was not mediated by mood.

#### Out-Group Defense

**FIGURE 3:**
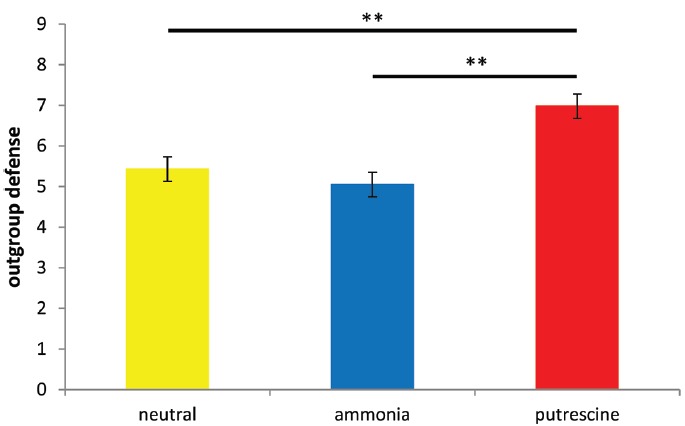
**Mean scores on the worldview defense scale for all three conditions (Experiment 4).** Higher scores reflect greater hostility toward the target. Asterisks denote two groups differ at **p < 0.005.

As predicted, we found a significant effect of scent prime on defensiveness toward the author of the essay, *F*(2,66) = 11.83, *p* < 0.001, η^2^ = 0.26 (see Figure 3). *Post hoc* analyses found that putrescine led to greater hostility (*M* = 6.98, SD = 1.42) compared to ammonia (*M* = 5.05, SD = 1.54) and the neutral conditions (*M* = 5.43, SD = 1.30; both *p*s < 0.005). There was no significant difference between the ammonia and control conditions, *p* > 0.6.

Experiment 4 supported the hypothesis that non-conscious exposure to putrescine evoked defensive responses toward an out-group member, and this effect was not due to conscious awareness of the scents, mood, or to the motivation to escape the aversive scent primes^3^. Although these results suggest that the scent primes elicited an odor percept (non-consciously), future studies may wish to control the precise intensities of the stimulus odors that are presented (e.g., using an olfactometer).

## General Discussion

This research was designed to test the hypothesis that putrescine could serve as a warning signal that mobilizes protective responses to deal with threats. In four experiments, we found support for this idea: conscious and non-conscious exposure to putrescine elicited distancing and defensive reactions (e.g., fight and flight responses). Putrescine increased vigilance (Experiment 1), heightened the accessibility of escape- and threat-relevant cognitions (Experiment 3), and increased the speed participants walked away from the location of the scent (Experiments 2 and 3). Experiment 4 created a situation where immediate escape was not likely and gave participants the opportunity to evaluate an out-group member. Subtle exposure to putrescine produced greater defensiveness toward the out-group member, suggesting an aggressive readiness in participants (Cesario et al., 2010). As a whole, the findings indicate that even brief exposure to putrescine mobilizes threat management responses designed to cope with environmental threats.

These are the first results to show that a specific chemical compound (putrescine) can be processed as a threat signal. Thus far, nearly all the evidence for threat chemosignals has come from those that are transmitted by body sweat (de Groot et al., 2012; Pause, 2012). Moreover, these are among the first studies that show that a specific chemical compound can cause overt behavior in humans (Wysocki and Preti, 2004). Furthermore, an advantage of isolating putrescine in threat management processes is that it may help in determining which sensory and brain pathways are involved in chemosensory threat detection and processing. For instance, research suggests that the central nucleus of the amygdala projects to the midbrain periaqueductal gray, the hypothalamus and the brainstem, which together coordinate to prepare fight-or-flight responses to threatening stimuli (Misslin, 2003). We speculate that putrescine activates a similar neurological pathway. Future research could include physiological measurements (e.g., systolic blood pressure, heart rate) to test the thesis that the observed effects of putrescine are modulated by processes originating in the sympathetic nervous system.

An important direction for future research will be to understand the precise nature of the threat produced by putrescine (e.g., microbial, predatory). Our view is that putrescine is relevant to both of these domains, though the immediate context should determine which type of threat is more primary. Recent work on TAARs has the potential to shed light on some of these mechanisms, as the activation of different receptors may function to detect specific threats, such as predators and pathogens (Li and Liberles, 2015; Pérez-Gómez et al., 2015). In addition, this research suggests that cadaverine (a compound with a similar chemical structure as putrescine; both are diamines) activates a similar pathway and produces similar escape and avoidance responses (Hussain et al., 2013; Oliveira et al., 2014) in animals. Thus, we render it likely that cadaverine evokes a similar threat response as putrescine (see Li and Liberles, 2015).

It would also be interesting to examine how putrescine detection affects sensitivity to particular types of threat and whether it produces elevated responses to certain stimuli more than others (e.g., fear- vs. disgust-based sensitivities). For instance, further research could elucidate how putrescine activates sensory acquisition (typically associated with fear experiences) and sensory rejection (associated with disgust) processes (Susskind et al., 2008), and whether exposure to putrescine augments physiological responses (e.g., heart rate, pupil dilation) that typically co-occur with adaptive responses to threats. This type of research would benefit from including individual differences in both disgust and fear sensitivity (Haidt et al., 1994; Garfinkel et al., 2014). By the same token, future work could clarify whether putrescine elicits discrete emotions (e.g., fear vs. disgust) or less specific affective states associated with negative valence and high arousal (see also Smeets and Dijksterhuis, 2014; Li and Liberles, 2015). Our findings, which showed that responses to putrescine were automatic, occurred after various lengths of delay (Experiments 1–3) and when presented at sub-threshold levels (Experiment 4), suggested that conscious evaluations are not at the heart of the observed responses to putrescine. This is consistent with our theorizing and ample work showing that chemosensory cues influence psychological and physiological operations outside of conscious awareness (for extended reviews, see Sela and Sobel, 2010; Smeets and Dijksterhuis, 2014). However, we hasten to add that more research is needed to specify the exact nature of the effects produced by the sub-threshold priming of putrescine, for instance, by varying the exposure times to putrescine, the delay after the primes, and the intensity of the putrescine stimulus.

Another important question is how specific threat management responses develop. Within non-olfactory sensory channels, for example, there may be an innate bias for humans to detect certain biologically-relevant stimuli as threatening, such as the sight of snakes and spiders (Ohman and Mineka, 2001). Although controversial in human research, some work suggests that responses to chemosensory stimuli are innate (Dielenberg et al., 2001; Misslin, 2003; Hussain et al., 2013). For instance, Soussignan et al. (1997) showed that soon after birth, butyric acid (a malodorous scent) evoked disgust reactions in neonates, a finding they claim is consistent with an innate predisposition toward ecologically-relevant scents. To test for the possibility of innate biases toward threatening chemosensory cues, it would be interesting to examine whether putrescine triggers facial expressions associated with fear or disgust in infants. In fact, research indicates that adults do not habituate so readily to the scent of putrescine emitted from rotting flesh (Roberson et al., 2008), suggesting that there might be a bias to respond warily to it.

Although the innateness of responses to chemosignals is still controversial, humans’ ability to incorporate learned information into cultural practices is beyond question (Boyd and Richerson, 2005). Consequently, the magnitude of specific chemosensory threat responses could be different in cultures where people are exposed to putrescine more frequently. Likewise, reactions to putrescine may differ between cultures with different burial practices (e.g., embalming practices, the duration before burial). These factors should remind us that the context is critical to how people react to putrescine. How olfactory information modulates other sensory inputs (Zhou et al., 2012) is no doubt central to whether it will be interpreted as threatening.

One alternative theoretical perspective of our findings on the effects of putrescine is terror management theory (TMT; Greenberg et al., 1994). According to this theory, reminders of death are regulated by a “cultural anxiety buffer” that consists of beliefs and values that imbue life with meaning and the promise of immortality. Interestingly, TMT argues that a great deal of the darker side of human behavior (e.g., aggression, out-group prejudice, religious intolerance) stems from the need to maintain and defend the integrity of this cultural anxiety buffer, due to its vital role in managing existential angst. In this view, putrescine could function as a reminder of mortality, and subsequently elicit similar defensive processes, as activated by reminders of death. We do not rule out this possibility, but render it unlikely that chemosensory threats trigger the same type of processes as those that originate from the unique human ability to reflect on the conundrum of life and death (Landau et al., 2007). Nevertheless, examining whether putrescine can be used as a subtle reminder of death, and whether it influences cultural beliefs, values, and practices, would open up fascinating directions of research.

Most research has shown that humans process threats either visually or audibly, while other animals inhabit the inaccessible world of scents. At the same time, we know that humans are guided by many of the same olfactory processes, especially when they involve fitness-relevant information. We believe that by identifying putrescine as one of these signals, a further understanding of its mechanisms can shed light on more general processes that modulate chemosensory signaling and threat management responses.

### Conflict of Interest Statement

The authors declare that the research was conducted in the absence of any commercial or financial relationships that could be construed as a potential conflict of interest.

## Footnotes

1 In line with previous research (Wheatley and Haidt, 2005), we pilot-tested a so-called “fart spray” along with skatole, indole, and ammonia, for suitability as an aversive control condition. These ratings are presented in Table 1. As can be seen, ammonia and fart spray were rated similarly to putrescine on all three dimensions of repugnance, familiarity, and intensity, whereas indole and skatole diverged from putrescine on at least one dimension. A disadvantage of fart spray, however, is that we could not ascertain its precise chemical compounds—its manufacturers were reluctant to disclose this information.

2 Because previous research has shown that men and women tend to walk at different speeds (Chumanov et al., 2008), the results of Experiments 2 and 3 included gender as a covariate. In addition, we analyzed the results of Experiments 2 and 3 with gender as a separate factor and this did not alter the significance of the results.

3 When the amount of time participants took to complete the questionnaire was used as a covariate, the results remained significant, *F*(2,65) = 13.13, *p* < 0.001, η^2^ = 0.29.
